# From Dengue to Demyelination: A Case of Brainstem Neuromyelitis Optica Spectrum Disorder Following Dengue Fever

**DOI:** 10.7759/cureus.87900

**Published:** 2025-07-14

**Authors:** Adnan Karajgi, Sai K Upadhyayula, Ramchandra Prabhu, Akshita Chintakindi

**Affiliations:** 1 Internal Medicine, Kempegowda Institute of Medical Sciences, Bangalore, IND

**Keywords:** aquaporin-4 antibody, brainstem syndrome, dengue fever, neuromyelitis optica spectrum disorder (nmosd), post-infectious demyelination

## Abstract

Neuromyelitis optica spectrum disorder (NMOSD) is a rare, autoimmune astrocytopathy characterized by demyelination and predominantly affects the optic nerves and spinal cord. Brainstem involvement, although less common, can lead to atypical presentations that complicate timely diagnosis. We present the case of a previously healthy woman in her early 40s who developed a rapidly progressive and ultimately fatal brainstem syndrome following recovery from dengue fever. She initially experienced intractable hiccups a few days after resolution of dengue-related symptoms. This progressed to dysphagia, excessive drooling, slurred speech, and ultimately respiratory compromise. Neurological examination revealed bulbar dysfunction, and she required ventilatory support within days of symptom onset. MRI of the brain demonstrated T2/FLAIR hyperintensities in the dorsal medulla and inferior olivary nucleus, with additional lesions in the frontal and perirolandic cortices. Notably, there was no evidence of optic neuritis or myelitis. Cerebrospinal fluid analysis was positive for aquaporin-4 immunoglobulin G (AQP4-IgG), confirming the diagnosis of NMOSD.

Despite receiving high-dose intravenous methylprednisolone, plasma exchange, and rituximab, her condition continued to deteriorate. She developed aspiration pneumonia and sepsis, ultimately resulting in death. This case underscores the importance of considering NMOSD in the differential diagnosis of isolated brainstem syndromes, particularly in the post-infectious context. Arboviral infections such as dengue may act as immune triggers in susceptible individuals. In endemic regions, early recognition of atypical neurological presentations and timely initiation of immunotherapy are essential to improve patient outcomes and reduce mortality from fulminant autoimmune demyelinating disease.

## Introduction

Neuromyelitis optica spectrum disorder (NMOSD) is a rare autoimmune demyelinating condition of the central nervous system (CNS), primarily characterized by recurrent episodes of optic neuritis and transverse myelitis [1]. The identification of aquaporin-4 immunoglobulin G (AQP4-IgG) antibodies has significantly improved the diagnostic framework, distinguishing NMOSD as a separate entity from multiple sclerosis (MS) [1]. While the optic nerves and spinal cord are typically affected, NMOSD can also involve the brainstem, diencephalon, and cerebral hemispheres [2].

Infections have long been implicated as potential triggers for autoimmune conditions, and emerging data support a post-infectious mechanism in some NMOSD presentations. Arboviruses such as Zika virus and Japanese encephalitis virus have been associated with immune-mediated neurological syndromes [3]. More recently, dengue virus - an endemic flavivirus in tropical and subtropical regions - has been increasingly linked to neurological complications [4]. Documented post-dengue complications include Guillain-Barré syndrome (GBS), acute disseminated encephalomyelitis (ADEM), and transverse myelitis. However, NMOSD remains an exceedingly rare consequence in this context [5].

We present, to our knowledge, the first reported case of AQP4-IgG positive NMOSD manifesting as acute brainstem syndrome following dengue fever. This case highlights the importance of maintaining clinical vigilance for non-opticospinal NMOSD phenotypes and underscores the need for early diagnosis and aggressive immunotherapy in rapidly progressive disease.

## Case presentation

We report the case of an Indian woman in her early 40s, with no significant medical history, who presented with a 10-day history of progressive neurological deterioration, beginning with intractable hiccups the day after being discharged from a six-day hospitalization for NS1-antigen positive dengue fever. During her initial admission, the patient presented with fever, myalgia, and thrombocytopenia, consistent with classical dengue fever. Her symptoms resolved with supportive care under observation. After discharge, she developed dysphagia, excessive salivation, severe speech impairment, and eventually respiratory compromise. On readmission, she was in acute respiratory distress, requiring supplemental oxygen followed by continuous positive airway pressure (CPAP). Subsequently, she was transferred to the intensive care unit, where an elective tracheostomy was performed to maintain airway protection, and she continued to breathe spontaneously on CPAP support.

Neurological assessment on readmission revealed a Glasgow Coma Scale of E4V5M6. Higher mental functions were preserved. However, cranial nerve examination revealed profound dysfunction: absent bilateral gag and cough reflexes, flaccid tongue with reduced tone and power, drooling, severe dysarthria, and upbeat gaze-evoked nystagmus. Lower motor neuron involvement was noted in cranial nerves VII through XII. Fundoscopy was normal, and there were no visual complaints. Initially, motor strength in all four limbs was Medical Research Council (MRC) grade 4/5 with preserved tone, bulk, and reflexes. Plantar responses were bilaterally flexor. However, within a few days, she demonstrated worsening quadriparesis, with the MRC grade dropping to 3/5 and diminished deep tendon reflexes. Auscultation revealed bilateral crackles, raising concerns for aspiration.

Laboratory evaluation revealed leukocytosis with neutrophilic predominance, prompting empirical initiation of broad-spectrum antibiotics. Serological testing for HIV, hepatitis B and C, VDRL, Varicella, anti-nuclear antibody (ANA), and anti-MOG (myelin oligodendrocyte glycoprotein) antibodies were negative. Cerebrospinal fluid (CSF) analysis was unremarkable: normal protein and glucose, no pleocytosis, absence of oligoclonal bands, and a negative acid-fast bacillus test. Repeat dengue serology was not obtained during the second admission. Electrolyte panel was normal except for a brief hypokalemic episode, which was corrected. Liver function tests, renal parameters, ECG, echocardiogram (ejection fraction 58%), and abdominal ultrasonography were all within normal limits.

**Figure 1:**
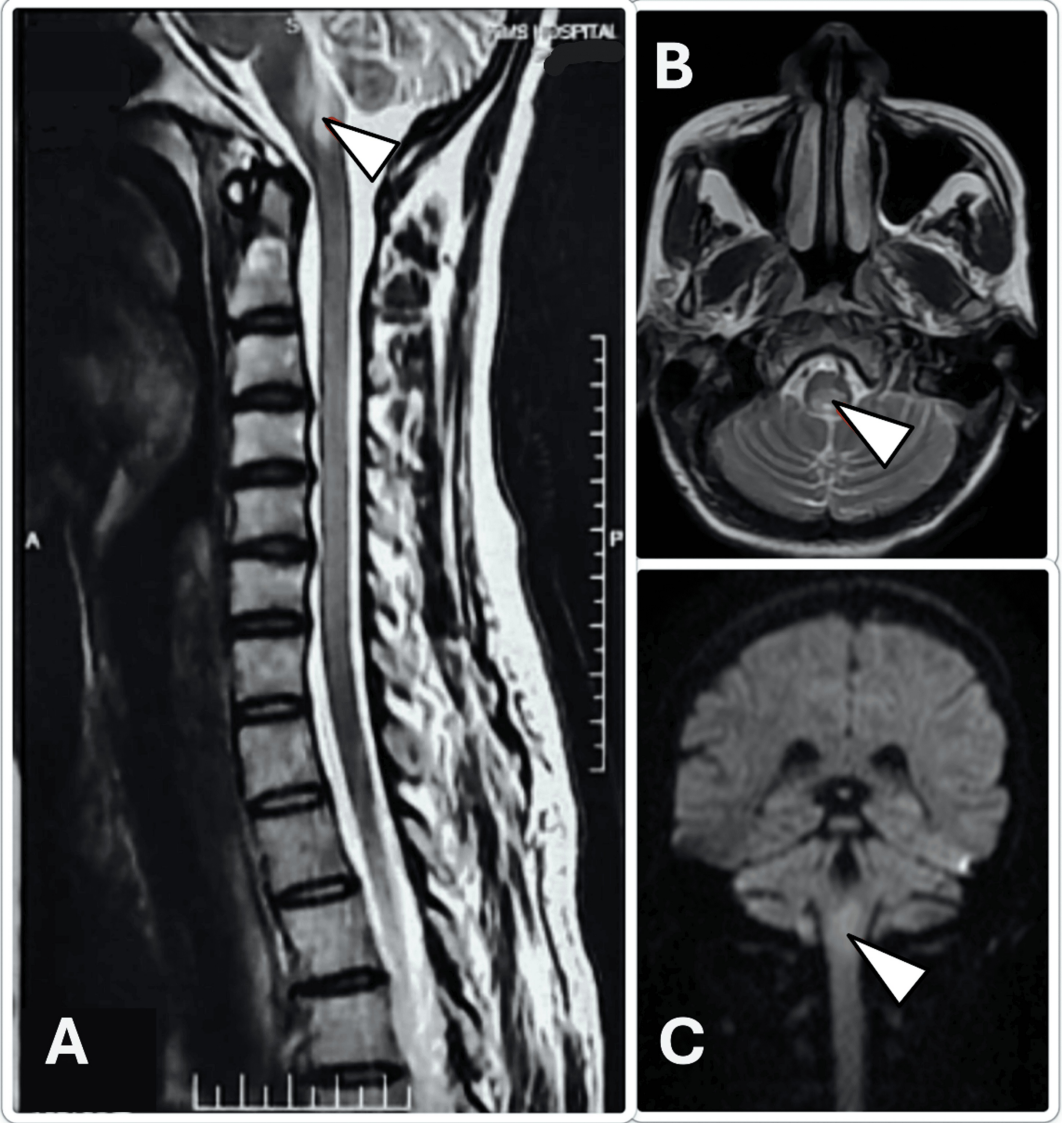
MRI of the brain with a screening of cervical spine showing FLAIR hyperintensities (white arrows) involving various regions of the dorsal medulla, cerebellum, pyramidal decussation, and inferior olivary nucleus. A. Sagittal view. B. Axial view. C. Coronal view. MRI, magnetic resonance imaging; FLAIR, fluid-attenuated inversion recovery

Magnetic resonance imaging (MRI) of the brain revealed diffusion restriction and T2/FLAIR hyperintensities in the bilateral inferior cerebellar peduncles, pyramidal decussation, medulla, inferior olivary nucleus, left perirolandic subcortical white matter, right frontal gyri, and right hippocampus (Figure 1), findings indicative of widespread brainstem involvement. Importantly, there were no optic nerve or spinal cord lesions, making this a highly atypical NMOSD presentation. Clinically, the presence of upbeat nystagmus and bulbar signs pointed toward dorsal medullary and area postrema involvement, characteristic of brainstem NMOSD. CSF AQP4-IgG returned strongly positive, establishing the diagnosis.

**Figure 2:**
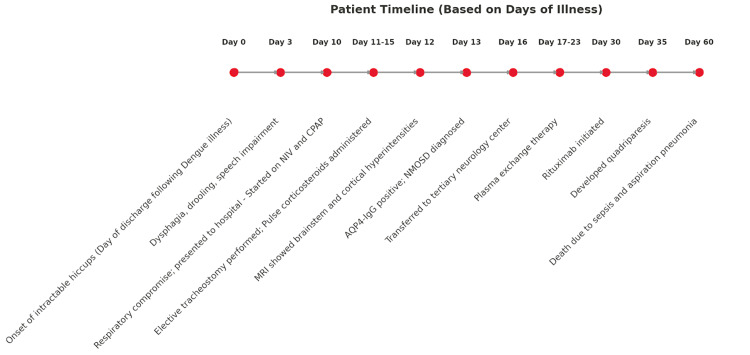
Course of events following the onset of illness AQP4-IgG, anti-aquaporin-4 immunoglobulin G; CPAP, continuous positive airway pressure; MRI, magnetic resonance imaging; NIV, non-invasive ventilation; NMOSD, neuromyelitis optica spectrum disorder

A five-day course of high-dose intravenous methylprednisolone (1 g/day) yielded no clinical improvement. She was referred to a tertiary neurology center where plasma exchange therapy was given for a week (three cycles, on alternate days), followed by rituximab therapy (1g IV, two doses, two weeks apart). Despite this escalation, her condition continued to decline, culminating in quadriparesis approximately one month after symptom onset. She ultimately died from aspiration pneumonia and septicemia due to multidrug-resistant organisms (Figure 2).

## Discussion

Dengue fever, caused by dengue virus, has increasingly been associated with neurological complications during both the acute and post-infectious phases. These include CNS involvement (e.g., encephalitis. encephalopathy), neuro-ophthalmological syndromes (e.g., optic neuritis), immune-mediated demyelination (e.g., NMOSD, GBS, ADEM, myelin oligodendrocyte glycoprotein antibody disease [MOGAD], transverse myelitis), and myopathies [4]. NMOSD, particularly AQP4-IgG positive variants, remains an exceptionally rare complication post-dengue [5].

AQP4-IgG antibodies target aquaporin-4 on astrocytic foot processes, leading to complement-mediated astrocyte injury and demyelination [6]. Viruses can trigger such autoimmunity through peripheral mechanisms such as molecular mimicry and bystander activation [3]. Although dengue virus can invade the CNS, neurological complications are more often due to systemic immune activation while the virus remains outside the CNS. Antibodies likely reach the brain via a disrupted blood-brain barrier, supported by inconsistent CSF detection of dengue virus in neurological cases [7].

Neuroimaging can offer important clues for differentiating NMOSD from other demyelinating diseases such as ADEM and MOGAD, particularly when involving the brainstem. In NMOSD involving the brainstem, lesions typically localize to the dorsal medulla, area postrema, peri-ependymal surfaces of the fourth ventricle, and periventricular brainstem regions. These lesions are often longitudinal and confluent and involve the central gray matter. In contrast, ADEM typically presents with widespread bilateral, asymmetric lesions involving the subcortical and deep white matter, basal ganglia, and thalami, often with incomplete ring enhancement and less involvement of the brainstem. MOGAD-associated lesions may be large, fluffy, and more often cortical or subcortical, with a higher likelihood of complete recovery and better treatment response [8]. Equally important is distinguishing NMOSD from other mimicking conditions such as MS and GBS, which share overlapping clinical features but differ significantly in management and prognosis. For instance, MS often presents with shorter spinal cord lesions and oligoclonal bands in CSF, while GBS is a peripheral demyelinating disorder that necessitates a different treatment approach altogether. Encephalitis, often infectious or autoimmune, may involve altered mental status and seizures, features less typical in NMOSD [9].

Acute brainstem syndrome occurs in 10-30% of NMOSD cases [10], and although optic or spinal involvement often follows, the natural history post-dengue remains unclear [11]. Brainstem involvement, especially of the bulbar and medullary centers, increases the risk of aspiration, respiratory failure, and death [12,13]. Our patient’s steroid-refractory course and rapid deterioration despite plasma exchange and rituximab align with severe brainstem NMOSD phenotypes [10]. Access to newer biologics such as eculizumab, satralizumab, or inebilizumab may improve outcomes in such refractory cases but was unavailable in our setting [14].

## Conclusions

This case highlights a unique and fatal presentation of AQP4-IgG-positive NMOSD as acute brainstem syndrome following dengue fever - an association not previously described. It underscores the need to include NMOSD in the differential diagnosis of rapidly progressive brainstem syndromes, particularly in the post-infectious context. Early testing for AQP4-IgG, detailed neuroimaging, and prompt escalation to advanced immunotherapies are essential to improving outcomes. As global dengue prevalence increases, clinicians should maintain a high index of suspicion for post-infectious autoimmune complications. Further research is warranted to define the pathophysiology and optimal treatment strategies for post-dengue NMOSD.
